# Surviving and Thriving—Shifting the Public Health Response to HIV-Exposed Uninfected Children: Report of the 3rd HIV-Exposed Uninfected Child Workshop

**DOI:** 10.3389/fped.2018.00157

**Published:** 2018-05-30

**Authors:** Amy L. Slogrove, Renaud Becquet, Ellen G. Chadwick, Hélène C. F. Côté, Shaffiq Essajee, Rohan Hazra, Valériane Leroy, Mary Mahy, Maurine Murenga, Jacqueline Wambui Mwangi, Laura Oyiengo, Nigel Rollins, Martina Penazzato, George R. Seage, Lena Serghides, Marissa Vicari, Kathleen M. Powis

**Affiliations:** ^1^Department of Paediatrics & Child Health, Stellenbosch University, Cape Town, South Africa; ^2^Bordeaux Population Health Research Center, Institut National de la Santé et de la Recherche Médicale, University of Bordeaux, Bordeaux, France; ^3^Feinberg School of Medicine, Northwestern University, Chicago, IL, United States; ^4^Department of Pathology and Laboratory Medicine, The University of British Columbia, Vancouver, BC, Canada; ^5^Senior Advisor in HIV, UNICEF, New York, NY, United States; ^6^Maternal and Pediatric Infectious Disease Branch, Eunice Kennedy Shriver National Institute of Child Health and Health and Human Development, National Institute of Health, Bethesda, MD, United States; ^7^French Institute of Health and Medical Research, Institut National de la Santé et de la Recherche Médicale, Paris, France; ^8^Strategic Information Department, UNAIDS, Geneva, Switzerland; ^9^Lean on Me Foundation, Nairobi, Kenya; ^10^The National Empowerment Network of People Living with HIV in Kenya, Maragoli, Kenya; ^11^Ministry of Health, Nairobi, Kenya; ^12^HIV Department, World Health Organization, Geneva, Switzerland; ^13^Department of Epidemiology, Harvard T.H. Chan School of Public Health, Boston, MA, United States; ^14^Toronto General Hospital Research Institute, University Health Network, Toronto, ON, Canada; ^15^Department of Immunology and Institute of Medical Sciences, University of Toronto, Toronto, ON, Canada; ^16^Women's College Research Institute, Toronto, ON, Canada; ^17^Collaborative Initiative for Paediatric HIV Education and Research, International AIDS Society, Geneva, Switzerland; ^18^Departments of Internal Medicine and Pediatrics, Masschusetts General Hospital, Boston, MA, United States; ^19^Department of Immunology and Infectious Diseases, Harvard T.H. Chan School of Public Health, Boston, MA, United States

**Keywords:** HIV-exposed uninfected, HEALTH OUTCOMES RESEARCH, developmental outcomes, survival, health monitoring

## Abstract

Great gains were achieved with the introduction of the United Nations' Millennium Development Goals, including improved child survival. Transition to the Sustainable Development Goals (SDGs) focused on surviving, thriving, and transforming, representing an important shift to a broader public health goal, the achievement of which holds the promise of longer-term individual and societal benefits. A similar shift is needed with respect to outcomes for infants born to women living with HIV (WLHIV). Programming to prevent vertical HIV transmission has been successful in increasingly achieving a goal of HIV-free survival for infants born to WLHIV. Unfortunately, HIV-exposed uninfected (HEU) children are not achieving comparable health and developmental outcomes compared with children born to HIV-uninfected women under similar socioeconomic circumstances. The 3rd HEU Child Workshop, held as a satellite session of the International AIDS Society's 9th IAS Conference in Paris in July 2017, provided a venue to discuss HEU child health and development disparities. A summary of the Workshop proceedings follows, providing current scientific findings, emphasizing the gap in systems for long-term monitoring, and highlighting the public health need to establish a strategic plan to better quantify the short and longer-term health and developmental outcomes of HEU children.

## Introduction

Scale up of antiretroviral treatment (ART) to pregnant and breastfeeding women living with HIV (WLHIV) has had a remarkable impact on reducing infant acquisition of HIV, yielding a 47% reduction between 2010 to 2016 (1). While this is a welcomed public health success, many observational studies have shown that HIV-exposed uninfected (HEU) children are not achieving equivalent health, developmental, and survival outcomes to that of similarly situated HIV-unexposed uninfected (HUU) children born to HIV-uninfected women (2–9). With the shift from the focus of the United Nations' Millennium Development Goals on child survival to the Sustainable Development Goals (SDGs) focused on surviving, thriving and transforming (10), the milestone for children born to WLHIV needs to extend beyond merely HIV-free survival to a framework that incorporates HIV-free survival with attainment of optimal health and developmental outcomes.

Following on the first two HEU Child Workshops that explored differences in immune development, infectious morbidity risks, neurodevelopment, and optimizing research methods for HEU research, the 3rd HEU Workshop considered the benefits and risks of maternal ART for the HEU child and how to establish long-term evaluation of these (11). The 3rd Workshop was conducted as a satellite session at the International AIDS Society's 9th IAS Conference on HIV Science held in Paris, France in July 2017, and was sponsored by the World Health Organization, the Collaborative Initiative for Paediatric HIV Education and Research, and the Pediatric HIV/AIDS Cohort Study. Herein, we present a summary of workshop proceedings, concluding with the need for a strategic plan to better quantify the short and longer-term health and developmental outcomes of HEU children through well-designed cohort studies as well as national and global monitoring systems.

## Discussion

Provisional 2017 UNAIDS Spectrum estimates of the size of the HEU child population were shared for the first time by Dr. Mary Mahy (UNAIDS). Spectrum estimates indicate that globally in 2016 there were approximately 1.4 million infants born to WLHIV, 1.25 million of whom remained HIV-uninfected and more than 1 million exposed *in utero* or postnatally to antiretroviral drugs (ARVs). Cumulatively, as of 2016 approximately 18 million children under 15 years of age have been HIV-exposed *in utero* but are HIV-uninfected, about 50% of whom also have *in utero* and/or postnatal ARV exposure. While the benefits of treating maternal HIV disease are undeniable, in an HEU child population of this size it is essential to continuously and objectively evaluate the benefits and potential risks of early life exposure to both ARVs and HIV in order to identify the safest ARVs and to elucidate mechanisms for observed disparities for which potential interventions can be introduced.

### Quantifying benefits and risks to HEU children of maternal ART

Dr. Renaud Becquet (Bordeaux University) presented results from a collaborative individual patient meta-analysis with the objective to assess mortality risk in HEU children as well as to estimate the contribution of identified risk factors in mediating poor HEU child outcomes. More than 19,000 HEU children were included in this analysis that pooled data from 21 clinical trials and observational studies in Africa and Asia (12). Seventy five percent of included children were born in sub-Saharan Africa and 70% were born prior to 2005. Mortality occurred early at a median (interquartile range) of 113 (38–244) days with cumulative incidence [95% confidence interval (CI)] for mortality at 6, 12, and 24 months of 3.1% (2.8–3.3), 4.4% (4.1–4.7), and 5.5% (5.1–5.9), respectively. Maternal mortality was most strongly associated with HEU child mortality [adjusted hazard ratio (aHR) 11.1, 95% CI 8.3–15.0]. Low birth weight (aHR 2.87, 95% CI 2.47–3.35) and never breastfeeding (aHR 2.51, 95% CI 1.96–3.20) more than doubled the mortality hazard. HEU children of mothers on maternally-indicated ART had a 49% lower hazard of mortality (aHR 0.51, 95% CI 0.28–0.94) compared to children of mothers receiving no ARVs during pregnancy. Breastfeeding in combination with maternally indicated ART reduced HEU child mortality by 96% (aHR 0.04, 95% CI 0.03–0.07), when compared to children of mothers not on maternally-indicated ART and not breastfeeding. At a population level, maternal mortality accounted for 4.3% (95% CI 2.3–9.2) of HEU child mortality, never initiating breastfeeding 10.8% (95% CI 4.7–17.9), low birth weight 16.2% (13.1–19.2), and mothers not on ART 45.6% (19.1–63.9). In combination, these four factors accounted for 63.6% (95% CI 45.7–76.6) of all HEU child mortality, leaving approximately one third of HEU child mortality still unexplained. This analysis confirms that prior to universal ART, maternally-indicated ART and breastfeeding were of substantial survival benefit to HEU children. New studies are needed to understand, whether in the era of universal ART and safer breastfeeding, HEU child survival will be comparable to HUU children.

Dr. Lena Serghides (Toronto General Hospital Research Institute), shared findings from Canadian and Ugandan cohorts of pregnant WLHIV, as well as laboratory models illustrating hormonal dysregulation associated with ARV use during pregnancy. In Canadian WLHIV, use of protease inhibitor (PI)-based regimens in pregnancy was associated with decreased progesterone and increased estradiol levels (13). In a Ugandan cohort of pregnant WLHIV, those randomized to a PI-based regimen had higher estradiol levels, while those randomized to an efavirenz-based regimen had lower estradiol levels compared to WLHIV not yet initiated on ART (14, 15). This demonstrates that ARV classes differentially influence two key hormones required to maintain a healthy pregnancy. Dr. Serghides presented her team's mouse experiments, demonstrating that decreased progesterone following PI-based ART in pregnancy was associated with lower fetal weight. This adverse effect was partially ameliorated with progesterone supplementation in pregnancy (13). Dr. Serghides shared experiments, using both human placental tissue and her lab's mouse model, elucidating mechanistic pathways responsible for this hormonal dysregulation (16).

Dr. Hélène Côté (University of British Columbia) presented findings from the pan-Canadian “Children and Women: Antiretrovirals and Markers of Aging” (CARMA) cohort and on behalf of the CARMA study team. She started with a focus on autism spectrum disorder (ASD). The Canadian population prevalence of ASD averages 1.5%, yet a Canadian cohort of HEU children was found to have an ASD prevalence of 5.7%. Dr. Côté and colleagues compared plasma mitochondrial DNA (mtDNA) levels between HEU children enrolled in CARMA and matched HUU children. HEU children diagnosed with ASD had the highest mtDNA content, followed by HEU children without ASD, HUU children with ASD, and HUU children without ASD. Higher mtDNA content was significantly associated with both HEU status and ASD status (17). In this analysis, perinatal ARV exposure was not a significant predictor of mtDNA content, although the study was not powered or designed to detect such a difference.

Dr. Côté also shared a CARMA study of neurodevelopmental outcomes, comparing results from HEU and matched (1:3) HUU children born between 1990 and 2012 (18). In multivariable analysis, controlling for time to follow-up, infant sex, and maternal smoking and/or substance use, the odds of an HEU child having one or more neurodevelopmental disorders (including ASD, emotional disturbances, hyperkinetic syndrome, developmental delay, intellectual disability, or epilepsy) was 1.5 (95% CI 1.2–2.5) times greater than an HUU child. Additional adjustment for preterm birth reduced the risk of having one or more neurodevelopment disorders by 20%, highlighting the strong association between preterm birth and neurodevelopmental outcomes, and the additional risk this adverse birth outcome places on HEU children (19–22). The work from the CARMA cohort further suggest that HEU children with *in utero* exposure to triple ARV regimens may have a lower risk of adverse neurodevelopmental outcomes, a trend requiring validation in larger cohorts and in high HIV burden settings.

The benefits of maternal ART undeniably outweigh the potential risks of *in utero* ARV exposure for HEU children. However, as in the examples presented at the Workshop, laboratory studies and small cohort studies have raised concerns about potential long-term implications of ARV and HIV exposure. These concerns and risks are still not well-defined and will remain so without investment in systems for long-term evaluation of HEU child outcomes.

### Establishing systems for long-term evaluation of HEU child outcomes

The Workshop audience heard presentations from Dr. Ellen Chadwick (Northwestern University) providing an overview of the highly-resourced United States (US)-based Surveillance Monitoring of ART Toxicities (SMARTT) cohort study structure and from Dr. Mary Mahy (UNAIDS) on challenges of and opportunities for establishing national and global HEU child monitoring.

The SMARTT cohort includes over 3,000 HEU infants, children, and adolescents in active follow-up, along with their caregivers, from 22 sites in the United States, including Puerto Rico. The novel trigger-based design employed by the SMARTT study provides an efficient way to identify associations between *in utero* exposures to specific ARV drugs and adverse events, after controlling for social, maternal, and infant characteristics (23). Under the trigger-based design, study-wide adverse events represent signals that “trigger” in depth epidemiologic and clinical investigations in select cases. Event domains monitored in the SMARTT study include growth, metabolic outcomes, mitochondrial function, neurologic outcomes including microcephaly and seizures, neurodevelopmental outcomes, chemistry, hematological and other laboratory adverse events, and hearing impairment. SMARTT visits are conducted throughout infancy, childhood, and adolescence. Dr. Chadwick shared some of the challenges with the SMARTT cohort, issues germane across all settings regardless of resources, including the difficulty in enrolling truly “matched” HUU infant, child and adolescent comparator groups, and the fact that HUU comparator groups need to be sufficiently large to achieve adequate power to detect rare adverse events, an expensive proposition.

While monitoring of the HEU child population is warranted, Dr. Mahy identified national and global challenges to this. Ideally designed monitoring systems measure progress toward targets over time or while implementing interventions expected to change outcomes. Monitoring systems for children living with HIV have matured over time. However, similar systems do not yet exist for HEU children. Some, but not all national programs, have sufficiently robust health systems with child health and/or immunization visit data that could be disaggregated, including by maternal HIV status, maternal ARV use in pregnancy and during breastfeeding, and child HIV exposure and infection status, facilitating monitoring of HEU child outcomes. However, even where health systems could support disaggregation of data to facilitate HEU child health outcome monitoring, longer term monitoring through health information systems is challenging as children get older and are no longer in regular contact with the healthcare system. Additionally, child well-being measures change over time and while it will be essential to compare HEU to HUU child outcomes, this will require integration of HEU child outcome monitoring within the broader child health monitoring systems. In the current era of universal ART, if maternal ART, maternal survival, and breastfeeding represent the major protective factors against HEU child mortality, we would expect to see HEU child mortality declining over the coming years as universal ART and uptake of safer breastfeeding continue to expand in countries experiencing generalized HIV epidemics. Monitoring of HEU child mortality trends at a national level in selected high HIV burden countries can provide evidence of whether maternal ART and safer breastfeeding are sufficient to ameliorate excess mortality risk in HEU children at a population level.

The SMARTT cohort study team has found that some WLHIV are hesitant to disclose their HIV status to their HEU child/adolescent. To address this, the study's caregiver and young adult Community Advisory Board, in collaboration with SMARTT investigators, developed disclosure materials specifically designed for use by caregivers, with separate materials for study staff. These tools assess caregiver readiness for disclosure and provide caregiver support for disclosure. Without disclosure to the HEU adolescent, long-term follow-up of adult health and survival consequences following *in utero* exposure to HIV and ARVs is precluded, a point of tension that was raised again during the panel discussion. From the individual perspective, WLHIV expressed the critical need to engage with communities in a process of learning together how to track long term HEU child outcomes, using an approach that affords confidentiality, prevents stigma, and ensures children enjoy their childhood. Parents considering disclosure of HEU status to their adolescent children require time to prepare both themselves and their children for the process. The issue of “stigma by association” for HEU children was expressed by panel members, who highlighted the importance of “mainstreaming” any programs to optimize and track HEU child outcomes into health programming for all children.

## Conclusion

The scale up of ART to pregnant and breastfeeding women living with HIV is one of the most remarkable public health achievements of the past decade, resulting in substantial reductions globally in infant perinatal and breastfeeding acquisition of HIV. This effort was driven, in part, by the impetus of the Global Plan toward the elimination of new HIV infections among children by 2015 and keeping their mothers alive (24). This program has now evolved into the “Start Free, Stay Free, AIDS Free” framework which promotes acceleration of action to prevent and treat HIV in children and adolescents within a life cycle approach (25). The value of this framework for action, particularly in setting public health goals, is imperative for strategically mapping out health infrastructure focus and investments. Furthermore, the framework implemented in response to the HIV/AIDS epidemic has demonstrated that access to high-quality data permits the establishment of ambitious, measurable, and time-bound targets that have been used to track progress and foster accountability. Yet no framework currently exists to support the rapidly expanding HEU child population. HEU children, having evaded HIV infection, no longer fall clearly within HIV programming and opportunities to support the HEU child population in the broader early childhood development framework are still being explored.

There is clearly a need to achieve consensus and develop a strategic plan for a new set of long-term cohort evaluation systems to identify subtle but possibly significant effects of HIV and ARV exposure on HEU children that individual studies are not able to identify or evaluate confidently. With the current emphasis on SDG number 17.18, calling for enhanced capacity building in developing countries to increase the availability of high-quality, timely, and reliable data disaggregated by characteristics relevant in national contexts, an opportunity exists to upgrade routine monitoring in child health and incorporate HEU children into the existing child health and SDG monitoring agenda in HIV high burden countries.

## Author contributions

AS and KP jointly prepared the initial manuscript with RB, EC, HC, SE, RH, VL, MarM, MauM, JW, LO, NR, MP, GS, LS, and MV all critically reviewing the manuscript and providing important intellectual edits.

### Conflict of interest statement

The authors declare that the research was conducted in the absence of any commercial or financial relationships that could be construed as a potential conflict of interest.

## Abbreviations

aHR: adjusted hazard ratio
ART: antiretroviral treatment
ARV: antiretroviral
ASD: autism spectrum disorder
CARMA: Children and Women: Antiretrovirals and Markers of Aging
CI: confidence interval
HEU: HIV-exposed uninfected
mtDNA: mitochondrial DNA
HUU: HIV-unexposed uninfected
PI: Protease Inhibitor
SDG: Sustainable Developmental Goals
SMARTT: Surveillance Monitoring of ART Toxicities
UBC: University of British Columbia
WLHIV: Women Living with HIV.

## References

[1] Joint United Nations HIV/AIDS Programme Ending AIDS Progress Towards the 90-90-90 Targets - Global AIDS Update 2017. Geneva: UNAIDS (2017) Available online at: http://www.unaids.org/sites/default/files/media_asset/Global_AIDS_update_2017_en.pdf

[2] le Roux SM Abrams EJ Nguyen K Myer L. Clinical outcomes of HIV-exposed, HIV-uninfected children in sub-Saharan Africa. Trop Med Int Health (2016) 21:829–45. 10.1111/tmi.12716 27125333

[3] Evans C Humphrey JH Ntozini R Prendergast AJ. HIV-Exposed uninfected infants in zimbabwe: insights into health outcomes in the pre-antiretroviral therapy era. Front Immunol. (2016) **7**:190. 10.3389/fimmu.2016.00190 27375613 PMC4893498

[4] Brennan AT Bonawitz R Gill CJ Thea DM Kleinman M Useem J et al. A meta-analysis assessing all-cause mortality in HIV-exposed uninfected compared with HIV-unexposed uninfected infants and children. AIDS (2016) 30:2351–60. 10.1097/QAD.0000000000001211 27456985

[5] Powis KM Smeaton L Ogwu A Lockman S Dryden-Peterson S van Widenfelt E et al. Effects of *in utero* antiretroviral exposure on longitudinal growth of HIV-exposed uninfected infants in Botswana. J Acquir Immune Defic Syndr. (2011)56:131–8. 10.1097/QAI.0b013e3181ffa4f5 21124227 PMC3023002

[6] Hofer CB Keiser O Zwahlen M Lustosa CS Frota AC de Oliveira RH et al. *In utero* exposure to antiretroviral drugs: effect on birth weight and growth among HIV-exposed uninfected children in Brazil. Pediatr Infect Dis J. (2016)35:71–7. 10.1097/INF.0000000000000926 26741583 PMC4705846

[7] Slogrove AL Goetghebuer T Cotton MF Singer J Bettinger JA. Pattern of infectious morbidity in HIV-exposed uninfected infants and children. Front Immunol. (2016) **7**:164. 10.3389/fimmu.2016.00164 27199989 PMC4858536

[8] Afran L Garcia Knight M Nduati E Urban BC Heyderman RS Rowland-Jones SL. HIV-exposed uninfected children: a growing population with a vulnerable immune system? Clin Exp Immunol. (2014) 176:11–22. 10.1111/cei.12251 24325737 PMC3958150

[9] Ezeamama AE Kizza FN Zalwango SK Nkwata AK Zhang M Rivera ML et al. Perinatal HIV status and executive function during school-age and adolescence: a comparative study of long-term cognitive capacity among children from a high HIV prevalence setting. Medicine (2016) **95**:e3438. 10.1097/MD.0000000000003438 27124032 PMC4998695

[10] United Nations Development Programme From the MDGs to Sustainable Development for All - Lessons from 15 Years of Practice. (2016). Available online at http://www.undp.org/content/undp/en/home/librarypage/sustainable-development-goals/from-mdgs-to-sustainable-development-for-all.html

[11] Slogrove AL Archary M Cotton MF. Optimizing research methods to understand HIV-exposed uninfected infant and child morbidity: report of the second HEU infant and child workshop. Front Immunol. (2016) **7**:576. 10.3389/fimmu.2016.00576 27999576 PMC5138183

[12] Arikawa S Rollins N Jourdain G Humphrey J Kourtis AP Hoffman I et al. Contribution of maternal ART and breastfeeding to 24-month survival in HIV-exposed uninfected children: an individual pooled analysis of African and Asian studies. Clin Infect Dis. (2017). 66:1668–77. 10.1093/cid/cix1102 29272387 PMC5961296

[13] Papp E Mohammadi H Loutfy MR Yudin MH Murphy KE Walmsley SL et al. HIV protease inhibitor use during pregnancy is associated with decreased progesterone levels, suggesting a potential mechanism contributing to fetal growth restriction. J Infect Dis. (2015) 211:10–8. 10.1093/infdis/jiu393 25030058 PMC4264589

[14] Balogun KA Guzman Lenis MS Papp E Loutfy M Yudin MH MacGillivray J et al. Elevated levels of estradiol in human immunodeficiency virus-infected pregnant women on protease inhibitor-based regimens. Clin Infect Dis. (2018) 66:420–7. 10.1093/cid/cix761 29020282 PMC5850422

[15] McDonald CR Conroy AL Gamble JL Papp E Hawkes M Olwoch P et al. Estradiol Levels are altered in human immunodeficiency virus-infected pregnant women randomized to efavirenz-versus lopinavir/ritonavir-based antiretroviral therapy. Clin Infect Dis. (2018) 66:428–36. 10.1093/cid/cix772 29136115 PMC5850641

[16] Papp E Balogun K Banko N Mohammadi H Loutfy M Yudin MH et al. Low prolactin and high 20-alpha-hydroxysteroid dehydrogenase levels contribute to lower progesterone levels in HIV-infected pregnant women exposed to protease inhibitor-based combination antiretroviral therapy. J Infect Dis. (2016) 213:1532–40. 10.1093/infdis/jiw004 26740274 PMC4837912

[17] Budd M Sampson L Bowes J Calli K Lewis M Cote H et al Elevated mitochondria DNA content in HIV-exposed uninfected children with autism. In Conference on Opportunistic Infections and Retroviruses. Boston, MA (2016).

[18] Piske M Budd MA Qiu AQ Maan EJ Sauve LJ Alimenti A et al Long-term developmental outcomes and in utero antiretroviral exposure to HIV-exposed uninfected (HEU) children born to mothers living with HIV in British Columbia (BC), Canada. In International AIDS Society Conference on HIV Science (Paris) (2017).

[19] Luu TM Rehman Mian MO Nuyt AM. Long-term impact of preterm birth: neurodevelopmental and physical health outcomes. Clin Perinatol. (2017)44:305–14. 10.1016/j.clp.2017.01.003 28477662

[20] Sucksdorff M Lehtonen L Chudal R Suominen A Joelsson P Gissler M et al. Preterm birth and poor fetal growth as risk factors of attention-deficit/ hyperactivity disorder. Pediatrics (2015)136:e599–608. 10.1542/peds.2015-1043 26304830

[21] Arpino C Compagnone E Montanaro ML Cacciatore D De Luca A Cerulli A et al. Preterm birth and neurodevelopmental outcome: a review. Childs Nerv Syst. (2010) **26:**1139–49. 10.1007/s00381-010-1125-y 20349187

[22] Dong Y Yu JL. An overview of morbidity, mortality and long-term outcome of late preterm birth. World J Pediatr. (2011) 7:199–204. 10.1007/s12519-011-0290-8 21822987

[23] Williams PL Seage GRIII Van Dyke RB Siberry GK Griner R Tassiopoulos K et al. A trigger-based design for evaluating the safety of in utero antiretroviral exposure in uninfected children of human immunodeficiency virus-infected mothers. Am J Epidemiol. (2012) 175:950–61. 10.1093/aje/kwr401 22491086 PMC3390009

[24] Joint United Nations HIV/AIDS Programme,. Global Plan Towards the Elimination of New HIV Infections among Children by 2015 Keeping Their Mothers Alive. Geneva: UNAIDS (2011). Available online at: http://www.unaids.org/sites/default/files/media_asset/20110609_JC2137_Global-Plan-Elimination-HIV-Children_en_1.pdf

[25] Joint United Nations HIV/AIDS Programme Start Free, Stay Free, AIDS Free. Geneva: UNAIDS (2015) Available online at: http://www.unaids.org/sites/default/files/media_asset/Stay_free_vision_mission_En.pdf

